# Profiling of serum metabolome of breast cancer: multi-cancer features discriminate between healthy women and patients with breast cancer

**DOI:** 10.3389/fonc.2024.1377373

**Published:** 2024-04-04

**Authors:** Katarzyna Mrowiec, Julia Debik, Karol Jelonek, Agata Kurczyk, Lucyna Ponge, Agata Wilk, Marcela Krzempek, Guro F. Giskeødegård, Tone F. Bathen, Piotr Widłak

**Affiliations:** ^1^ Center for Translational Research and Molecular Biology of Cancer, Maria Sklodowska-Curie National Research Institute of Oncology, Gliwice, Poland; ^2^ Department of Circulation and Medical Imaging, The Norwegian University of Science and Technology, Trondheim, Norway; ^3^ Department of Public Health and Nursing, The Norwegian University of Science and Technology, Trondheim, Norway; ^4^ Department of Biostatistics and Bioinformatics, Maria Sklodowska-Curie National Research Institute of Oncology, Gliwice, Poland; ^5^ Department of Systems Biology and Engineering, Silesian University of Technology, Gliwice, Poland; ^6^ Clinic of Radiology and Nuclear Medicine, St. Olavs Hospital, Trondheim University Hospital, Trondheim, Norway; ^7^ 2nd Department of Radiology, Medical University of Gdansk, Gdansk, Poland

**Keywords:** biomarker, breast cancer, high-resolution mass spectrometry, metabolomics, multicancer signature, serum metabolome, The HUNT study

## Abstract

**Introduction:**

The progression of solid cancers is manifested at the systemic level as molecular changes in the metabolome of body fluids, an emerging source of cancer biomarkers.

**Methods:**

We analyzed quantitatively the serum metabolite profile using high-resolution mass spectrometry. Metabolic profiles were compared between breast cancer patients (n=112) and two groups of healthy women (from Poland and Norway; n=95 and n=112, respectively) with similar age distributions.

**Results:**

Despite differences between both cohorts of controls, a set of 43 metabolites and lipids uniformly discriminated against breast cancer patients and healthy women. Moreover, smaller groups of female patients with other types of solid cancers (colorectal, head and neck, and lung cancers) were analyzed, which revealed a set of 42 metabolites and lipids that uniformly differentiated all three cancer types from both cohorts of healthy women. A common part of both sets, which could be called a multi-cancer signature, contained 23 compounds, which included reduced levels of a few amino acids (alanine, aspartate, glutamine, histidine, phenylalanine, and leucine/isoleucine), lysophosphatidylcholines (exemplified by LPC(18:0)), and diglycerides. Interestingly, a reduced concentration of the most abundant cholesteryl ester (CE(18:2)) typical for other cancers was the least significant in the serum of breast cancer patients. Components present in a multi-cancer signature enabled the establishment of a well-performing breast cancer classifier, which predicted cancer with a very high precision in independent groups of women (AUC>0.95).

**Discussion:**

In conclusion, metabolites critical for discriminating breast cancer patients from controls included components of hypothetical multi-cancer signature, which indicated wider potential applicability of a general serum metabolome cancer biomarker.

## 1 Introduction

In women’s population breast cancer (BC) represents 25% of newly diagnosed cancer cases and about 15% of cancer-related deaths worldwide. In many developed countries, this cancer ranks first on the list of morbidity and mortality among all malignancies. Moreover, according to epidemiologic forecasts, both values will increase over the next decades (1). Therefore, intensive research is needed on the mechanisms of development of this very heterogenous malignancy (2) to clarify many aspects of its molecular biology further and identify biomarkers for risk assessment and early detection of this cancer (3).

Since 1920, when Otto Warburg noticed the accumulation of lactate in tumor tissue due to increased glucose consumption by aerobic glycolysis, cancer metabolomics has made great progress (4, 5). The metabolome combines information resulting from both endogenous processes and exogenous interactions, thus providing insight into cellular mechanisms and their modifications caused by a wide range of stimuli. In addition, metabolic changes are visible earlier than phenotypic ones, and their examination is possible in a quick and minimally invasive way (e.g., by determination in body fluids). Studying the profiles of metabolites enables the creation of so-called “metabolic fingerprints’’, i.e. changes in the metabolome characteristic of a specific state of the body. Numerous studies have been conducted to characterize cancer-related changes, using different cohorts and on various types of material (tissue, blood, etc.) (6, 7). Metabolic features of breast cancer were addressed in several reports, including information on cancer-related changes in the metabolism of amino acids, fatty acids, or glycerolipids (8–11). Unfortunately, reported results are ambiguous, hence the metabolic fingerprint for breast cancer and its specificity regarding other cancer types have yet to be fully characterized (12).

In the current study, we performed a quantitative analysis of metabolites present in serum samples of breast cancer patients and two cohorts of healthy women, which allowed us to identify differences in the metabolic profiles of healthy women and patients diagnosed with breast cancer. Moreover, women with three other types of solid cancers (colorectal cancer, head and neck cancer, and lung cancer) were included in the study, which revealed “multi-cancer” characteristics of certain metabolic features observed in patients with breast cancer.

## 2 Materials and methods

### 2.1 Characteristics of analyzed groups

The clinical material was collected at the Maria Sklodowska-Curie National Research Institute of Oncology, Gliwice Branch between 2010 and 2020. Blood samples were collected from women patients with breast (BC), colorectal (CC), head and neck (HC), and lung (LC) cancers before the start of cancer therapy. Two groups of healthy donors were included in the study: healthy volunteers living in the Silesia region, Poland (Ctr_P), recruited in the same period as cancer patients, and a subset of healthy women selected from participants of the HUNT2 study performed between 1995 and 1997 in the Trøndelag region, Norway (Ctr_N). The Trøndelag Health Study (HUNT) is a collaboration between HUNT Research Centre (Faculty of Medicine and Health Sciences, Norwegian University of Science and Technology NTNU), Trøndelag County Council, Central Norway Regional Health Authority, and the Norwegian Institute of Public Health (13). The latter set included women selected from a group of 450 healthy participants analyzed in a previously published study (14) to match the age of BC patients (see diagram in **Supplementary Figure S1**). Consequently, two independent cohorts of healthy controls were included: Ctr_P and Ctr_N. The characteristics of the study cohort are presented in **Table 1**. Peripheral blood was collected into a 5 mL BD Vacutainer Tube, incubated for 30 min at room temperature to allow clotting, and then centrifuged at 1000× g for 10 min to remove the clot. The serum was aliquoted and stored at −80°C before further processing. The study was conducted following the Declaration of Helsinki, and approved by the Ethics Committee of Maria Sklodowska-Curie National Research Institute of Oncology, Gliwice Branch (KB/493-53/10 and KB/430-84/20) and the Regional Committee for Medical and Health Research Ethics (REK#1995/8395 and REK#2017/2231). All participants provided informed consent indicating their voluntary participation.

**Table 1:** Characteristics of the study cohorts.

	Control (Poland)		Control (Norway)		Breast Cancer		Colorectal Cancer	Head & Neck Cancer	Lung Cancer
Abbreviation	Ctr_P		Ctr_N		BC		CC	HC	LC
N	95	35*	112	35*	112	35*	30	32	35
Age (years) mean [S.D.]	48.3 [6.5]	53.7 [3.6]*	49.3 [11.0]	63.0 [9.0]*	49.3 [11.0]	60.5 [7.4]*	64.8 [10.5]	59.4 [11.9]	65.2 [8.8]
Clinical stage									
I	–	–	–	–	0	0*	6	0	9
II	–	–	–	–	56	19*	11	2	8
III	–	–	–	–	49	14*	12	9	13
IV	–	–	–	–	7	2*	1	21	5

*Sub-cohort of controls and BC cases selected for comparison with other solid cancers to enable similar age distribution in all groups.

### 2.2 Quantitative high-resolution mass spectrometry

Quantitative analysis of metabolites for all serum samples was performed using the Absolute IDQ p400 HR kit (Biocrates Life Sciences AG, Innsbruck, Austria) following the procedure recommended by the producer (**Supplementary Data**: Protocol for metabolite detection and quantification by the Absolute IDQ p400 HR kit). This is a commercial assay with an automated workflow, whose quality, stability, and repeatability were validated in the international ring trial (15). Orbitrap Q Exactive Plus high-resolution mass spectrometer (Thermo Fisher Scientific, Waltham, MA, USA) and 1290 Infinity UHPLC (Agilent, Santa Clara, CA, USA) system was used to measure concentrations of selected metabolites (including amino acids, biogenic amines, hexoses, acylcarnitines, diglycerides, triglycerides, (lyso)phosphatidylcholines, sphingolipids, and cholesteryl esters) in 10 µl human serum. Samples were measured in batches designed to secure the same proportion of different groups with a randomized order of samples within each group. The obtained chromatograms and spectra were processed using Xcalibur 4.1. and MetIDQ DB110-2976 software (Biocrates Life Sciences AG) resulting in a matrix of concentrations of metabolites in µM. To control the quality of quantitative analyses the coefficient of variation (CV) of all Quality Control (QC) measurements for all metabolites was calculated (16).

### 2.3 HRMS data processing

The MS dataset contained measurements of the levels of 389 metabolites present in 416 samples. Firstly, detection and imputation of missing values were performed. According to the recommendations of Chen and coworkers (17) a threshold of 50% was adopted for values missing not at random (i.e., values below the limit of detection). In the case of data missing completely at random (i.e., generated as a result of the internal standard error), a threshold of 10% was adopted. In the first case, missing values were imputed by random numbers generated from normal distribution truncated to a segment between 0 and the median value of the limits of quantitation for all test plates. In the second case, missing values were imputed using the k-nearest neighbor approach (the nearest observed data were identified using a correlation distance metric, and the mean value of the three nearest neighbors was used based on measurements collected for the same group). Metabolites that were non-compliant with these criteria were excluded from further analyses. Finally, 284 metabolites were qualified for quantitative analysis, and the remaining 105 compounds were left for binary analysis, which statistically tests whether the absence/presence status of a metabolite is a group-related feature. In the next step, the data were transformed using the log base 2 function, and then the batch effect was corrected using an empirical Bayes method, assuming that samples measured using a single 96-well sample preparation plate represent one batch (18).

### 2.4 Statistical and bioinformatics analyses

The quantitative analysis of metabolites that differentiated BC cases (n=112) and either control group (n=95 or n=112 for Ctr_P and Ctr_N, respectively) was performed using the Mann–Whitney U test, and then the Benjamini–Hochberg procedure was performed to reduce the number of false positive results. To analyze metabolites that differentiated either control group (n=35 for both Ctr_P and Ctr_N) from BC (n=35), CC (n=30), HC (n=32), and LC (n=35) cases, the Kruskal−Wallis test, followed by the post-hoc Conover test for pairwise comparisons was implemented. All statistical hypotheses were tested at the 5% significance level. In addition, the “r” effect size was calculated according to the formula: r=z/square root of N (where z is the value of the test statistic and N is the total number of observations in two compared groups) with interpretation according to the Cohen’s criterion (small effect – |r|>0.1, medium effect – |r|>0.3, large effect – |r|>0.5) (19). Fisher’s exact test was applied for metabolites that did not qualify for quantitative analysis to determine if there was a nonrandom association between the absence/presence of metabolites and analyzed groups. Hierarchical clustering was performed to assess similarities between the analyzed groups. The median value of metabolite abundances for samples within a particular group was calculated (raw abundances were previously transformed to z-scores). Each analyzed group was characterized by a vector consisting of the calculated median value of metabolite abundances, and then similarities between groups were analyzed using agglomerative hierarchical cluster analysis with the Minkowski distance between pairs of observations and the average linkage clustering method. To assess the predictive quality of the multi-cancer signature for distinguishing breast cancer samples and controls, a classifier was constructed on a dataset containing samples not used for the signature selection (60 Ctr_P, 60 Ctr_N, 77 BC cases). A support vector machine (SVM) model with a radial kernel function was trained on half of the BC cases (n=39) and an equal number of Ctr_N controls, and tested on the remaining half of the BC cases (n=38) and an equal number of Ctr_P controls (see diagram in **Supplementary Figure S1**); this design enabled the validation of the universality of classification model using different populations of healthy women. The prediction quality on the test set was evaluated in terms of accuracy, sensitivity, specificity, positive and negative predictive value (PPV and NPV, respectively), and area under the receiver operating characteristic curve (AUC). To obtain a reliable estimation of classification quality, the procedures (sampling, training, and testing/validation) were repeated 500 times. All analyses were performed using the R Statistical Software (version 4.1.2, R Foundation for Statistical Computing, Vienna, Austria). The metabolic pathway enrichment analysis was performed using the MetaboAnalyst 5.0 platform for all quantitative data (https://www.metaboanalyst.ca/MetaboAnalyst/ModuleView.xhtml; last access October 6, 2023).

## 3 Results

### 3.1 HRMS-based analysis of serum metabolites revealed compounds discriminating between controls and breast cancer cases

**Figure 1:**
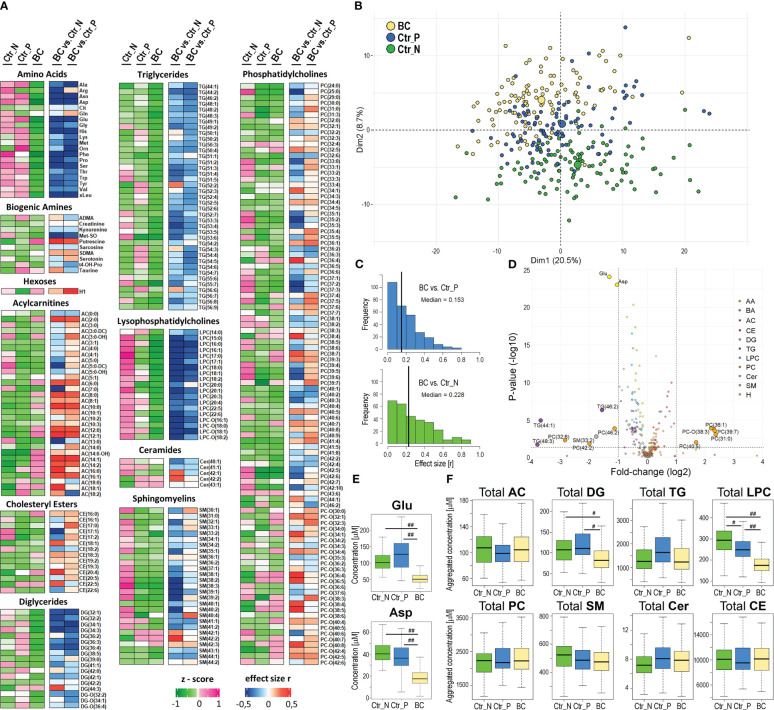
Characterization of the serum metabolome profile analyzed by mass spectrometry in breast cancer patients and healthy women. Panel **(A)** – Levels of metabolites in serum samples from 112 Norwegian and 95 Polish controls (Ctr_N and Ctr_P, respectively) and 112 cancer cases (BC); heatmap visualizes median levels of analyzed metabolites in each group (raw abundances were converted into z-scores) and magnitudes of differences between groups (quantified as “r” effect size). Panel **(B)** – Plot of the data in two dimensions of the first two principal components of PCA analysis to visually identify clusters; cases and controls are marked separately (large circles represent the average of each group). Panel **(C)** – The histograms for metabolites that showed the increased significance of differences between BC cases and either group of controls (vertical lines represent the median value of “r” effect sizes). Panel **(D)** – The volcano plot representing metabolites with significantly different concentrations between BC and Ctr_P; shown is the fold-change and corresponding p-value. Panel **(E)** – Concentrations of glutamic acid (Glu) and aspartic acid (Asp) in samples of BC cases and controls. Panel **(F)** – Differences in aggregated concentration of different classes of lipids between BC cases and controls. Boxplots represent minimum, lower quartile, median, upper quartile, and maximum; medium (|r|>0.3) or large (|r|>0.5) effect size is marked with one hash (#) or two hash (##) symbols, respectively; AC – acylcarnitines, DG – diglycerides, TG – triglycerides, LPC – lysophosphatidylcholines, PC – phosphatidylcholines, SM – sphingomyelins, Cer – ceramides, CE – cholesteryl esters.

Serum metabolite and lipid profiles were analyzed quantitatively by HRMS in a set of samples collected from breast cancer patients and two cohorts of healthy women living in Poland or Norway; three other types of solid cancer were also used for the comparison (baseline characteristics of the study groups are presented in **Table 1**; a similar age distribution was ensured between the compared groups). This approach enabled the detection of 389 metabolites, among which 284 compounds quantified in the majority of samples were used in quantitative analyses. The median value of concentrations of quantified compounds and the strength of differences between controls and BC cases are presented in **Figure 1A**. Good separation of cancer and control samples in the unsupervised Principal Component Analysis (PCA) was noted; moreover, a separation was also observed between both groups of control samples **(Figure 1B**, also see **Supplementary Figure S1A** for the results of the OPLS-DA analysis). There were 60 serum metabolites whose levels were significantly different between BC cases and Polish controls (medium or large effect size), including 49 metabolites downregulated and 11 upregulated in BC cases. On the other hand, there were 114 metabolites with levels significantly different between BC cases and Norwegian controls, including 88 downregulated and 26 upregulated in cancer samples (see **Supplementary Table S1** for details). Moreover, when the remaining set of 105 metabolites not qualified for quantitative analysis was tested for the absence/presence status, 5 metabolites (AC(15:0), AC(18:1), DG(38:0), PC(44:3), Cer(44:0)) were significantly under-represented in BC cases compared to Polish controls. On the other hand, 4 metabolites (AC(7:0), LPC-O(17:1), PC(35:0), PC(44:10)) were under-represented while 4 metabolites (AC(4:0-OH), spermidine, PC-O(44:5), PC-O(32:0)) were over-represented in BC cases compared to Norwegian controls (**Supplementary Table S2**).

Differences between (Polish) breast cancer cases and Norwegian controls in concentrations of serum metabolites were stronger than differences between cases and Polish controls (**Figure 1C**); the medians of the effect sizes equal 0.228 and 0.153, respectively. Partially different sets of metabolites that discriminated against BC cases and either group of controls were related to significant differences between both control groups. We found 85 compounds with different concentrations between Ctr_P and Ctr_N groups (**Supplementary Table S1**). Nevertheless, a large set of metabolites commonly differentiated BC cases from both control groups: there were 33 overlapped metabolites significantly downregulated in BC cases and 10 metabolites significantly upregulated in BC cases in comparison to both control cohorts (medium or large effect size; **Supplementary Table S1**). Importantly, the identification of the same features in two different cohorts of healthy women indicated a universal significance of the signature, which could be called the “breast cancer signature”. This signature included 13 amino acids, 12 lysophosphatidylcholines, and 6 diglycerides downregulated in BC cases as well as 9 acylcarnitines upregulated in BC cases. Noteworthy, the increased level of hexoses (incl. glucose) detected in cancer samples was significantly higher compared to Polish controls than compared to Norwegian controls (large and small effect size, respectively). The volcano plot in **Figure 1D** and **Supplementary Figure S3** illustrates the metabolites that showed the most robust differences between controls and BC cases. These included glutamic acid (Glu) and aspartic acid (Asp) whose concentration was markedly reduced in the serum of BC patients compared to both groups of healthy controls (**Figure 1E**). Moreover, assuming the potential functional redundancy of lipids from the same class, aggregated amounts of major classes of detected lipids were also compared (**Figure 1F**). We found that total levels of lysophosphatidylcholines (LPC) and diglycerides (DG) were markedly reduced in sera of breast cancer patients compared to both groups of controls (effect size r<-0.3). Similar total levels of lipid classes were observed in sera of healthy individuals from both control cohorts (except for lysophosphatidylcholines slightly upregulated in Norwegian controls).

### 3.2 HRMS-based analysis of serum lipids and metabolites revealed a common set of compounds that differentiated controls from breast cancer cases and three other types of solid cancers

**Figure 2:**
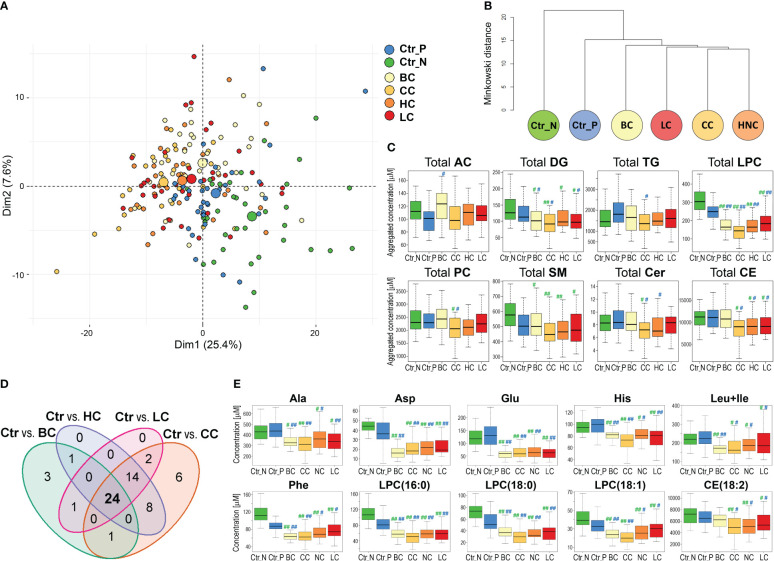
The serum metabolome features differentiate healthy women and patients with four types of solid cancers. Panel **(A)** – Plot of the data in two dimensions of the first two principal components of PCA analysis of samples of women diagnosed with breast (BC), colorectal (CC), head and neck (HC), and lung (LC) cancers, and healthy controls (large circles represent the average of each group). Panel **(B)** – Dendrogram of the hierarchical binary cluster tree of six groups based on the median value of metabolite concentrations; the height of each U represents the Minkowski distance between the two analyzed groups being connected within a cluster. Panel **(C)** – Differences in aggregated concentrations of the major classes of lipids between controls and four types of cancers; medium (|r|>0.3) or large (|r|>0.5) effect size of differences between control and particular cancer type is marked with one hash (#) or two hash (##) symbols, respectively (green marks on left and blue marks on right represent the significance of differences between cancer cases and Ctr_N or Ctr_P, respectively). Panel **(D)** – The Venn diagram showing the overlap of metabolites that discriminated four types of solid cancers from both groups of controls consistently (large and medium effect size). Panel **(E)** – Concentrations of selected metabolites that differentiated controls and four types of cancers (the effect size is marked as in Panel **C**).

Knowing serum metabolites that differentiated breast cancer patients from healthy controls, we aimed to check its potential specificity for this particular cancer compared to features of serum metabolome observed in other types of solid cancers. Therefore, additional groups of women patients diagnosed with either colorectal cancer (CC), head and neck cancer (HC), or lung cancer (LC) were included in the analysis. Assuming that the age of participants is a potential confounding factor in this type of study, smaller sub-cohorts of healthy controls and BC cases were selected to enable a similar age distribution in all groups (**Table 1**). The median value of concentrations of quantified compounds in all six groups and the strength of differences between controls and each cancer type are presented in **Supplementary Figure S4**. When clusters of samples were observed in the PCA analysis, both control groups were distinct from any cancer cases; Norwegian controls were the most dissimilar (**Figure 2A**; the analysis was based on all quantitated metabolites; also see **Supplementary Figure S1B** for the results of the OPLS-DA analysis). Similarly, unsupervised hierarchical clustering of “averaged group representatives” revealed the largest distance of controls from all cancer types (**Figure 2B**); CC cases and HC cases appeared the most similar. When the average strength of differences between controls and cancer cases were compared (based on the histogram of metabolites differentiating controls and cases with increasing effect size), the biggest effect was noted for CC and HC cases, while the effect was lower for BC cases (median value of the effect sizes equal to 0.157 and 0.259 for Ctr_P and Ctr_N, respectively) (**Supplementary Figures S5A, B**). Aggregated amounts of the major classes of lipids were also analyzed which revealed reduced levels of total serum lysophosphatidylcholines and diglycerides as a general cancer-related feature. On the other hand, certain lipid features characteristic of CC, HC, and LC were not observed in BC cases. These included downregulation of ceramides, sphingomyelins, and cholesteryl esters; the latter feature (i.e., downregulation of cholesteryl esters compared to both control groups) was statistically significant in all solid cancers except BC cases (**Figure 2C**).

Furthermore, we searched for specific metabolites whose serum levels differentiated both control cohorts from all cancer types (i.e., components of a hypothetical “multi-cancer” signature); cancer cases were analyzed against each control cohort separately (**Supplementary Table S3**). We found that 29 features discriminated between Polish controls and all four types of solid cancers (large and medium effect size); all but one (AC(14:1)) showed reduced concentration in cancer samples (**Supplementary Figure S5C**). On the other hand, 98 features discriminated between Norwegian controls and all four types of solid cancers, which included reduced total concentrations of lysophosphatidylcholines, diglycerides, and sphingomyelins in cancer samples (**Supplementary Figure S5D**). Importantly, when these two sets of metabolites common for all cancer types were combined, 24 overlapped features were revealed (**Figure 2D**). This common “multi-cancer signature” included 6 amino acids (Ala, Asp, Glu, His, Phe, Leu+Ile), 2 DGs, 2 TGs, and 13 LPCs (and total LPC level) (**Supplementary Table S4**).

This is noteworthy, that only a small fraction of compounds (5 metabolites) differentiating BC cases from both cohorts of controls did not belong to this multi-cancer signature. Hence, the major fraction of metabolites that differentiated controls and BC cases showed similar differences between controls and other types of solid cancers. On the other hand, a subset of features that discriminated both cohorts of controls from cancers LC, HC, and CC but not from BC cases was relatively large (14 features). These cancer-specific features that were missed in the case of breast cancer included reduced concentration of CE(18:2), which is the most abundant cholesteryl ester detected in the serum (in general, similar concentrations of cholesteryl esters were noted in BC cases and controls). Examples of metabolites that discriminated control and cancer samples, including components of a hypothetical multi-cancer signature, are presented in **Figure 2E**.

### 3.3 Breast cancer classifier based on serum metabolome components present in the multi-cancer signature

**Figure 3:**
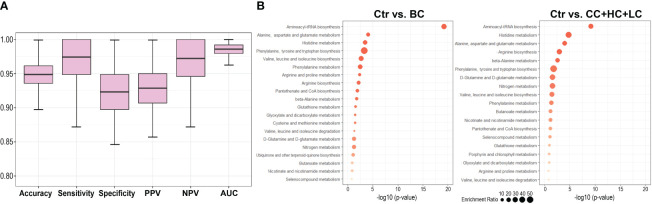
Comparison of the serum metabolite features characteristic of breast cancer patients and patients with other types of solid cancers. Panel **(A)** – Performance of breast cancer classifier built of features present in the multi-cancer signature. The classifier was trained and tested using Norwegian (Ctr_N) and Polish (Ctr_P) controls, respectively. Panel **(B)** – Metabolic pathways associated with compounds whose levels were different between controls and BC cases (left) or controls and other types of cancers (CC, HC, LC); shown are pathways with the most significant enrichment (size of a dot corresponds to the enrichment ratio).

Metabolites present in a hypothetical multi-cancer signature were used to test multicomponent binary classifiers discriminating breast cancer cases from healthy controls. Considering a high correlation of serum concentrations of 13 LPCs present in this signature, only an aggregated LPC level was included in the tested model. Therefore tested signature was composed of 11 features: Ala, Asp, Glu, His, Phe, Leu+Ile, DG(34:3), DG(36:4), TG(44:2), TG(46:2), and total LPC. Samples used for the identification of the multi-cancer signature were not used for training and testing of the classifier to avoid information leaks (scheme in **Supplementary Figure S1**). The classification model was trained using Norwegian controls and tested using Polish controls; this design strengthened the validation of the universality of the classification model. Five hundred repeats of the train/test procedure were implemented to assess the prediction power of the classifier. The indices of the classification model obtained during its vali-dation are presented in **Figure 3A**. In general, we found a very high prediction power of the resulting breast cancer classifier: sensitivity=0.97, specificity=0.92, and AUC=0.98. We concluded that a set of metabolites selected to differentiate healthy women from patients with four types of solid cancers (multi-cancer signature) classified an independent group of BC cases with very high precision.

### 3.4 Similar metabolic pathways were associated with breast cancer and three other types of solid cancers

The analysis of metabolic pathways associated with a set of 43 compounds whose levels differed between both groups of controls and BC cases (i.e., breast cancer signature) revealed several terms primarily related to amino acid metabolism. Pathway analysis was also performed with a set of 42 compounds whose levels differed between both groups of controls and three other types of cancer (CC, HC, and LC) not taking into account BC. Practically the same pathways were associated with sets of compounds differentiating controls from BC and compounds commonly differentiating controls from other types of cancers (**Figure 3B**); however, lipids that are not properly annotated in the used bioinformatics tool are not illustrated in either case. Nevertheless, the top-3 pathways were the same in both sets: “aminoacyl-tRNA bio-synthesis”, “alanine, aspartate, and glutamate metabolism”, and “histidine metabolism”, confirming the functional similarity of serum metabolites characteristic of BC and other types of solid cancers.

### 3.5 Possible confounding factors affecting the study

The age of participants is a strong factor affecting the serum concentrations of several metabolites, which is manifested by a positive correlation between age and concentration of lipids, particularly DGs (only concentrations of LPCs were age-independent) (14). However, the compared groups have a similar age distribution in the present study, which excluded age as a confounding factor during the comparison between controls and cancer cases. Nevertheless, we have performed additional analysis where differences between the BC cases and controls were analyzed separately in sub-cohorts of “younger” (<50 year-old) and “older” (≥50 year-old) women, which putatively mirrored their pre- and post-menopause statuses (**Supplementary Table S5**). This analysis revealed the same patterns of difference between the BC cases and both groups of controls (Ctr_P and Ctr_N) in either age-defined sub-cohorts, which indicated that features of cancer signature were age-independent). Another biological factor that putatively affects serum concentrations of metabolites is a fasting period before the blood collection, which was not controlled in the current study for cancer patients and Polish controls. However, based on data obtained from healthy participants of the HUNT2 study (14), we found that levels of lysophosphatidylcholines differentiating controls and cancer cases (e.g., LPC(16:0), LPC(18:0), and total LPC level) were not affected by fasting. Similarly, levels of amino acids essential for cancer classification (Asp, Glu, Phe), which were generally decreased in cancer samples, were barely affected by fasting (a slight increase with time of fasting could be noted) (**Supplementary Figure S6**), which further reduced the putative confounding significance of this factor for hypothetical cancer signature. Moreover, since the sample’s storage periods extended two years some metabolites might have been affected by long-term storage (20). Importantly, however, any changes induced by long-term storage were randomly distributed over the cases (BC, CC, HC, LC) and Ctr_P samples, which reduced the impact of this confounding factor on the observed differences between cancer patients and healthy controls. HUNT samples (Ctr_N) were stored for a longer time compared to Polish samples. However, proposed cancer signature included only compounds that jointly differentiated cancer samples from both groups of controls, which reduced the potential impact of differences in the storage period. Furthermore, measuring groups of samples as separate batches could result in differences strengthened by analytical factors such as instrumental drift. However, in the current study, all types of cancer and control samples were similarly distributed among sets/batches of measurements, and then potential batch effects among these sets were corrected during the data processing procedure.

## 4 Discussion

Metabolomics, which addresses the most dynamically changing system in the human body – the metabolome, represents an emerging opportunity for the understanding of human disease (6). The implementation of analytical approaches based on NMR and mass spectrometry for the detection and quantification of metabolites present in blood and other body fluids enabled the identification of multicomponent signatures that could be considered a goldmine of biomarkers of different cancers, including breast cancer (21, 22). However, though molecular features of breast cancer have been widely reported in the literature, the specificity of metabolic serum fingerprint for breast cancer has not been characterized comprehensively yet (12).

Here we applied an HRMS-based quantitative approach to compare metabolic profiles of serum from breast cancer patients and two cohorts of healthy women, which revealed a set of metabolites whose serum levels were significantly different be-tween cancer cases and controls. Cancer-related features were clearly distinguished despite significant differences between Polish and Norwegian cohorts of healthy women used as a control. Differences between the cohorts of healthy women are putatively related to differences in lifestyle-related factors, including diet and physical activity, since the potential influence of ethnic/genetic background was rather limited (however, due to lack of demographic details analysis of hypothetical lifestyle-related factors could not be performed) or changes in the metabolite concentrations during the extend sample storage (Norwegian samples collected in the frame of HUNT2 study were stored for a longer period than Polish cases and controls). We found that concentrations of most of the amino acids (Glu and Asn in particular), diglycerides, triglycerides, and lysophosphatidylcholines were generally decreased while concentrations of hexoses (incl. glucose), and certain acylcarnitines were increased in sera of breast cancer patients. A study that applied the earlier version of the quantitative MS-based platform than the one used in our study (the Biocrates p180 assay), revealed significantly reduced concentrations of several amino acids (Ala, Asn, Glu, His, Leu, Lys, Met, Orn, Phe, Thr, Trp, Val) and biogenic amines (kynurenine and Met-SO) in plasma of breast cancer patients (23), which was fully coherent with results of the current study. However, metabolic patterns of breast cancer reported in several other studies only partially overlapped with the present study. For example, multiplatform (NMR, LC-MS, and GC-MS) analysis of plasma metabolome performed in a group of Hispanic women with breast cancer revealed an increased concentration of several acylcarnitines (which was coherent with our study) but also triglycerides, and lysophosphatidylcholines (which was contrary to our study) (24). Another study based on the combination of LC-MS and GC-MS showed increased levels of Gln and acylcarnitines while decreased levels of lysophosphatidylcholines and amino acids in plasma of breast cancer patients, which was consistent with our study, yet levels of glucose were decreased in cancer (25). Such inconsistencies among reports could be due to different analytical platforms and different demography/pathology characteristics of studied cohorts (for example, the age of donors is a major confounding factor affecting the profile of metabolites in serum samples (14)). Nevertheless, the majority of studies showed reduced levels of amino acids in the plasma or serum of breast cancer patients (8, 23, 26). Moreover, similar to our report, increased levels of glucose (27, 28) were documented in other studies. Hence, though several differences among published reports exist, impaired metabolism of amino acids (manifested by their reduced serum/plasma concentrations) and glycolysis (manifested by increased concentration of glucose) appeared as general metabolic features observed in the blood of breast cancer patients.

Though major cancer-related changes in cellular metabolism are common for malignant cells (29, 30), specific differences could be observed when metabolic profiles of serum/plasma are compared among patients with different types of cancer. For example, differences in serum levels of certain amino acids and lipids were reported in patients with different leukemias (31). Here we observed several differences in serum metabolome patterns among four types of solid cancers. This could be exemplified by different serum lipid profiles: reduced levels of cholesteryl esters and phosphatidylcholines characteristic for patients with head and neck, lung, or colorectal cancer were not observed in patients with breast cancer. Nevertheless, a set of metabolites that significantly differentiated healthy controls from patients with all types of investigated solid cancers was identified. This set of metabolites comprised several amino acids (Ala, Asp, Glu, His, Leu, Ile, Phe) and lysophosphatidylcholines (including the most abundant LPC(16:0) and LPC(18:0)) with reduced serum concentrations in cancer patients. Hence, we concluded that metabolic features of serum that most markedly differentiated healthy women and breast cancer patients represent a set of metabolites common for women with different solid cancers, which could be considered as a “multi-cancer signature”. This conclusion was further confirmed because metabolites present in this signature could be used to build a specific breast cancer classifier that showed very high prediction power when validated using independent groups of women.

The characteristic feature of the hypothetical multi-cancer signature was the reduced serum level of amino acids and lipids, a phenomenon widely described in the literature. In general, reduced levels of metabolites in the serum of cancer patients could reflect their transfer from blood to the tumor site caused by their higher consumption by cancer cells, where they work as biosynthesis substrates, fatty acid carriers, energy sources, and/or signaling molecules. Increased uptake from blood and enhanced metabolism of amino acids is a hallmark of many cancers, including cancers addressed in the current study (32). Under different types of stress conditions, amino acids facilitate the survival and proliferation of cancer cells due to their essential role in nucleotide and protein synthesis or DNA methylation. Moreover, some amino acids function as precursors of polyamines or nitric oxide, as well as act as signaling molecules (33). Decreased level of circulating amino acids is linked to increased uptake by cancer cells due to overexpression of amino acid transporters, including *SLC1A5* (Gln uptake), *SLC1A4* (Ser uptake), *SLC7A5* (Leu, Ile, and Val uptake), or *SLC7A1* (Arg uptake) (4). Another tumor-related feature is generally reduced levels of serum lipids (so-called hypolipidemia) resulting from increased utilization of lipids by cancer cells (34). Few studies showed decreased serum levels of glycerides (35) and cholesterols (36) in breast cancer patients. However, the most characteristic feature observed in cancer patients is a reduced level of circulating lysophosphatidylcholines with a chain of palmitic, stearic, or oleic acids (LPC(16:0), LPC(18:0), and LPC(18:1), respectively). Reduced serum levels of LPCs putatively reflected their transfer to tumor tissue and higher consumption by cancer cells. This effect could also result from intensified conversion of LPC by autotaxin (ATX) to lysophosphatidic acid (LPA), since increased ATX expression was observed in different cancers, including breast cancer where it was linked to the promotion of metastasis (37). Moreover, lysophosphatidylcholine acyltransferase 1 (LPCAT1), which catalyzes the conversion of LPC to PC, is overexpressed in different tumors including breast cancer (38–40). Nevertheless, the metabolism of phosphatidylcholines is significantly disturbed in cancer cells and their increased incorporation into plasma membranes enhances proliferation and motility. Therefore, the changed serum levels of their precursors (e.g., choline) and/or derivatives (e.g., lysophosphatidylcholines) are considered promising cancer markers (41, 42). This is noteworthy that reduced levels of LPC(18:0) were associated with an increased risk of different tumors including breast, prostate, colorectal, and lung cancers (14, 43, 44). The reduced level of serum cholesterol was another cancer-related feature observed in our study, though this effect was milder in patients with breast cancer compared to other cancers. Nevertheless, reduced levels of cholesterol may result from the fact that cholesterol is a key precursor of estrogen (45). Moreover, cholesterol-reach LDLs impact the proliferation of breast cancer cells due to the overexpression of Akt and ERK pathway intermediates (46), and high expression of LDL receptors was detected in breast cancer cells (47). Furthermore, increased serum concentrations of acylcarnitines, compounds involved in lipid and energy metabolism (48), were also characteristic of cancer patients. Noteworthy, similar metabolic pathways were associated with sets of compounds characteristic of breast cancer and characteristic of other types of solid cancers. Therefore, one should assume that metabolites whose serum levels differentiated healthy women from patients with breast cancer and other solid tumors were undoubtedly associated with metabolic pathways generally impaired in cancer cells.

Concentrations of serum metabolites are markedly affected by several biological and preanalytical confounding factors. These confounders include but are not limited to age, fasting status, and extended sample storage which were considered in the present study. However, other pre-analytical factors related to sample processing (49, 50) not controlled in the current study may be present as the cohorts were collected in the frame of different studies. Hence, the significance of the multi-cancer signature proposed in our pilot study should be further validated in the independent prospective study involving cohorts matched regarding age and medical conditions other than cancer status, where samples are collected and processed in fully standardized and controlled conditions. Nevertheless, a major strength of our results is the discovery of cancer signatures obtained in comparison with two different cohorts of control samples, and revealing the overlap between serum metabolome signatures of breast cancer and other types of solid cancers.

## 5 Conclusions

The high-throughput metabolomics approach implemented in the current study revealed a set of serum metabolites that discriminated between healthy women and breast cancer patients. The identified breast cancer signature included metabolites associated with known cancer-related pathways. Despite some differences in serum metabolome profiles among women with different solid cancers, a common set of metabolic features that discriminated cancer patients from healthy controls was established. Noteworthy, metabolites critical for discriminating breast cancer patients from controls included components of a hypothetical multi-cancer signature, which indicates wider potential applicability of a general metabolic cancer biomarker after its comprehensive validation.

## Data availability statement

The original contributions presented in the study are included in the article/**Supplementary Material**. Further inquiries can be directed to the corresponding authors.

## Ethics statement

The studies involving humans were approved by Ethics Committee of Maria Sklodowska-Curie National Research Institute of Oncology, Gliwice Branch (KB/493-53/10 and KB/430-84/20) and the Regional Committee for Medical and Health Research Ethics (REK#1995/8395 and REK#2017/2231). The studies were conducted in accordance with the local legislation and institutional requirements. The participants provided their written informed consent to participate in this study.

## Author contributions

KM: Formal analysis, Investigation, Methodology, Writing – original draft. JD: Investigation, Writing – review & editing. KJ: Investigation, Methodology, Project administration, Writing – review & editing. AK: Data curation, Formal analysis, Methodology, Writing – review & editing. LP: Resources, Writing – review & editing. AW: Formal analysis, Writing – review & editing. MK: Formal analysis, Writing – review & editing. GG: Conceptualization, Writing – review & editing. TB: Conceptualization, Funding acquisition, Resources, Writing – review & editing. PW: Conceptualization, Funding acquisition, Supervision, Writing – review & editing.
