# Supplementary material for: Profiling of serum metabolome of breast cancer: multi-cancer features discriminate between healthy women and patients with breast cancer
Source: Front Oncol. 2024 Apr 4;14:1377373. doi: 10.3389/fonc.2024.1377373 (PMC11027565; doi:10.3389/fonc.2024.1377373)
Supplement: Supplementary file 1 [file DataSheet_1.pdf]

Supplementary Material

1 Supplementary Data: Protocol for metabolite detection and quantification by the Absolute IDQ p400 HR kit.

Samples were analyzed by a quantitative approach using a combined flow injection analysis (FIA) and liquid chromatography (LC) high-resolution mass spectrometry (HRMS) assay with the Absolute IDQ p400 HR kit (Biocrates Life Sciences AG, Innsbruck, Austria). The LC-HRMS method was used to quantify amino acids and biogenic amines while acylcarnitines, cholesterol esters, glycerophospholipids, glycerides, sphingolipids, and hexoses were assessed using FIA-HRMS. The kit provided quantitative measurements for 12 acylcarnitines, 21 amino acids, 21 biogenic amines, and the sum of hexoses. For the amino acids and biogenic amines, standard solutions for calibration curves at seven levels including isotopically labeled internal standards were provided for each metabolite. For the acylcarnitines and sum of hexoses, standards at one concentration were provided (one-point calibration using FIA). The rest of the metabolites (172 phosphatidylcholines, 24 lysophosphatidylcholines, 31 sphingomyelins, 9 ceramides, 43 acylcarnitines, 14 cholesteryl esters, 18 diglycerides, and 42 triglycerides) were measured semiquantitatively, e.g. standards with similar chemical properties as the targets were used (a version of one-point calibration).

Sample preparation was performed according to the detailed protocol that was provided with the kit. Briefly, the samples were prepared on the kit plate (all plates provided with the kit were in the 96-well format), by first adding stable isotope-labeled standards followed by 10 µL of the serum sample. The samples were then dried using an evaporator and subsequently derivatized by the addition of 50 µL of a 5% solution of phenylisothiocyanate (in water:ethanol:pyridine, 1:1:1) followed by incubation at room temperature for 20 min. The samples were then dried again, and extracted by the addition of 300 µL 5 mM ammonium acetate in methanol (MeOH) and shaking at 450 rpm for 30 min. The extracts were collected by centrifugation into the provided collection plate. For LC-HRMS analysis, 150 µL of the samples were transferred and diluted with 150 µL water on an empty plate, and for FIA-HRMS analysis, 250 µL of the FIA mobile phase (made by mixing 290 mL MeOH and a 10 mL ampule Biocrates FIA mobile phase additive) were added directly to the samples on the collection plate.

The extracts were analyzed using a 1290 Infinity UHPLC system (Agilent, Santa Clara, CA, USA) coupled to a high-resolution Q Exactive™ plus Orbitrap mass spectrometer (Thermo Fisher Scientific, Waltham, MA, USA) using electrospray ionization. The instrumental analysis was executed according to the guidelines from the kit manufacturer. In brief, the analysis was performed in the positive ionization mode for both LC-HRMS and FIA-HRMS, and the data were collected in the full-scan (MS1) mode. The chromatography was executed using Agilent Zorbax Eclipse XDB-C18 (3.5 µm) 3.0 x 100 mm column provided with the kit, with the injection volume 5 µL. Mobile phase A was 0.2% formic acid in water and mobile phase B was 0.2% formic acid in acetonitrile. The chromatographic program was 6 min, including a gradient from 0 to 95% B over 4 min, followed by washing (95% B) and equilibration (0% B). The flow rate was 0.8 mL/min and the column oven temperature was 50 °C. The FIA analysis was done by injecting 20 µL of the sample into the flow of the FIA program, lasting for 5 min, and pumping the FIA mobile phase (10 mL ampule Biocrates FIA mobile phase additive in 290 mL MeOH) at the flow rate 0.05 mL/min for the first 1.6 min, then increasing the flow rate to 0.2 mL/min for 1.2 min, and then back to 0.05 mL/min for the rest of the program.

2 Supplementary Figures

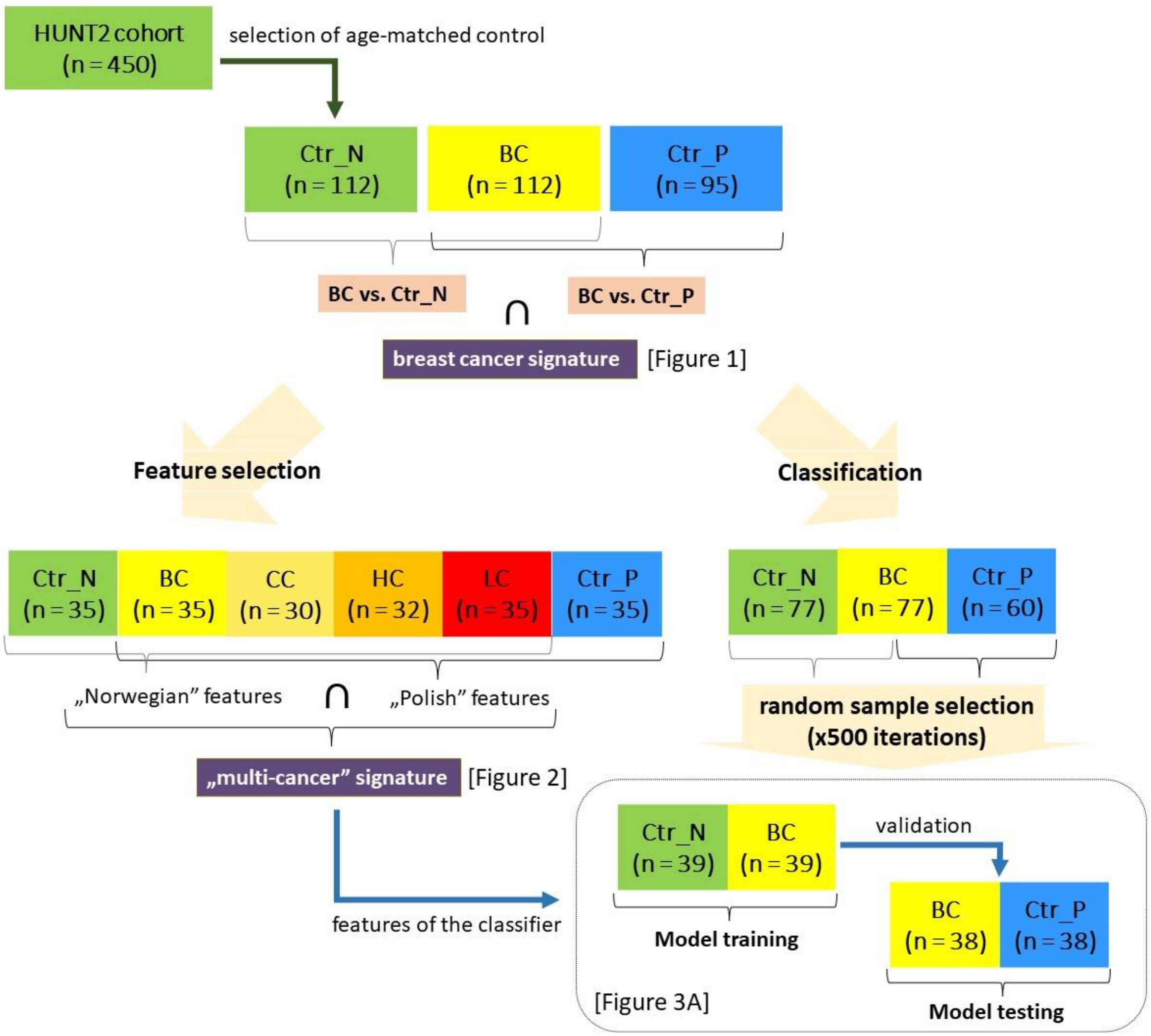

Supplementary Figure S1. Diagram of sample selection at different stages of analyses.

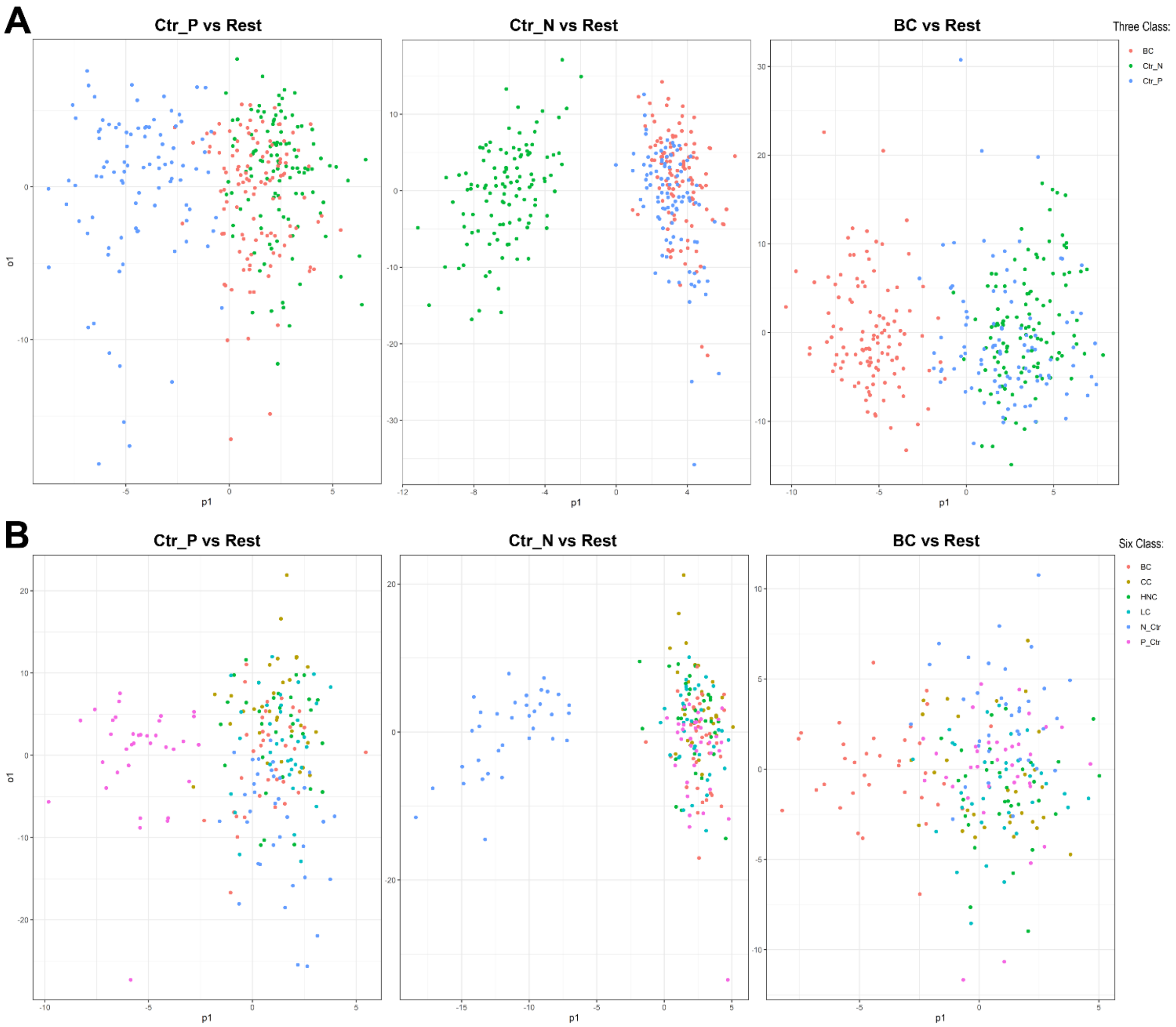

Supplementary Figure S2. Clustering of samples as illustrated in the OPLS-DA analysis. **Panel A** – One-versus-Rest comparisons among three groups: Ctr_P, Ctr_N, and BC. **Panel B** – One-versus-Rest comparisons among six groups: Ctr_P, Ctr_N, BC, CC, HC, and LC; selected figures represent comparisons of either Ctr_P, Ctr_N, or BC versus the rest of the groups.

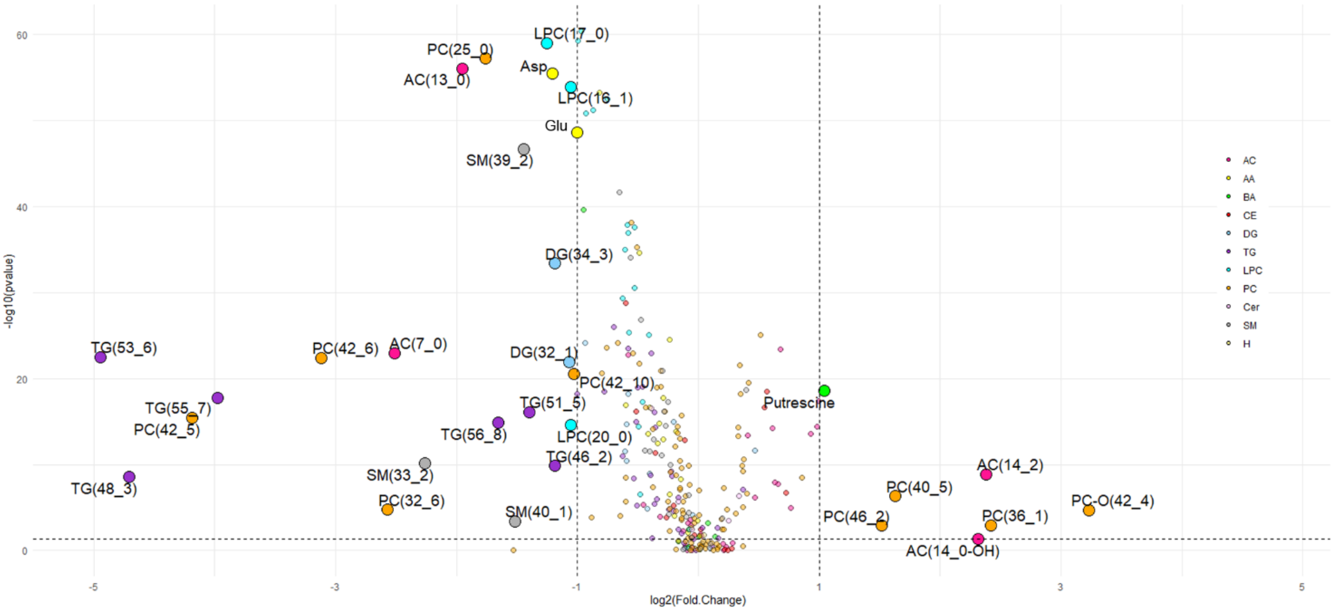

Supplementary Figure S3. The volcano plot represents metabolites with significantly different concentrations between BC cases and Ctr_N controls; shown are the fold-change and corresponding p-value.

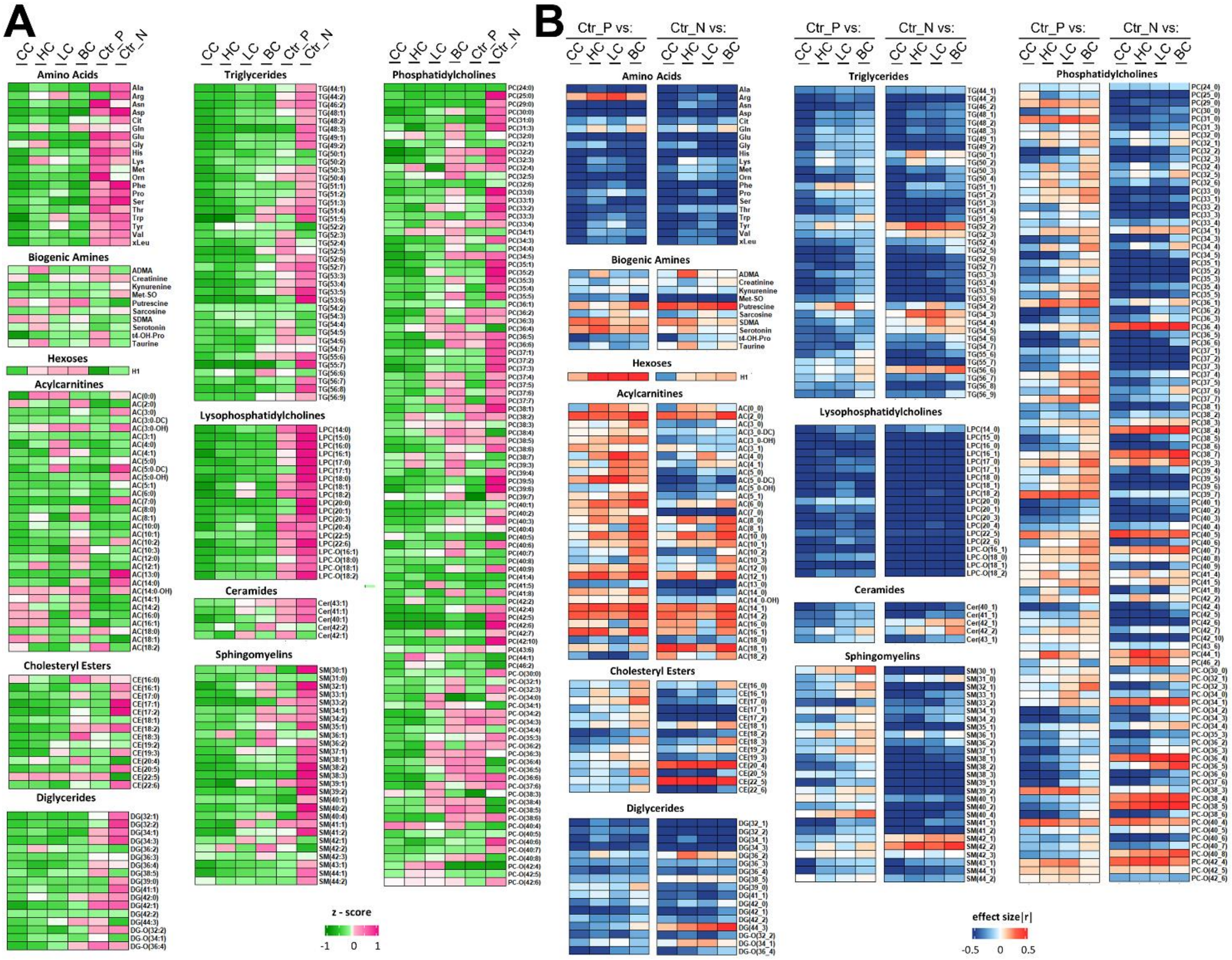

Supplementary Figure S4. Characterization of the serum metabolome profile analyzed by mass spectrometry in women diagnosed with colorectal (CC), head and neck (HC), lung (LC), and breast (BC) cancers, and healthy Polish (Ctr_P) and Norwegian (Ctr_N) controls. **Panel A** – Heatmap represents average levels of analyzed metabolites in each group of samples (raw abundances were converted into z-scores). **Panel B** – Heatmap represents the magnitude of differences between controls and cancer cases for each cancer type (quantified as “r” effect size); differences between cases and controls are shown separately for both groups of controls.

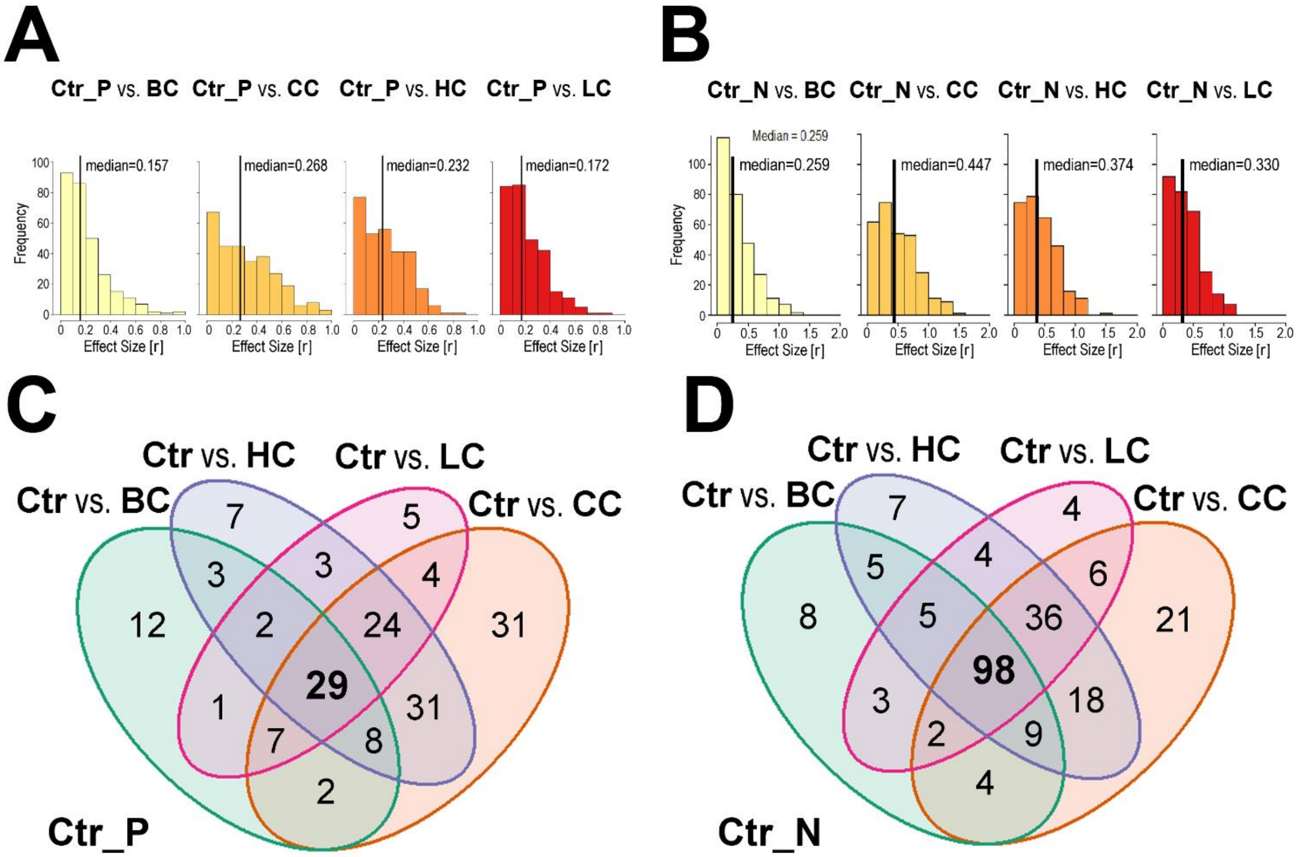

Supplementary Figure S5. Differences between cancer cases and healthy controls. The histograms for metabolites that showed the increased significance of differences between each cancer type and Ctr_P (**Panel A**) or Ctr_N (**Panel B**) controls (vertical lines represent the median value of "r" effect sizes). The Venn diagrams show the overlap of metabolites that discriminated four types of solid cancers (large and medium effect size) from Ctr_P (**Panel C**) or Ctr_N (**Panel D**) controls.

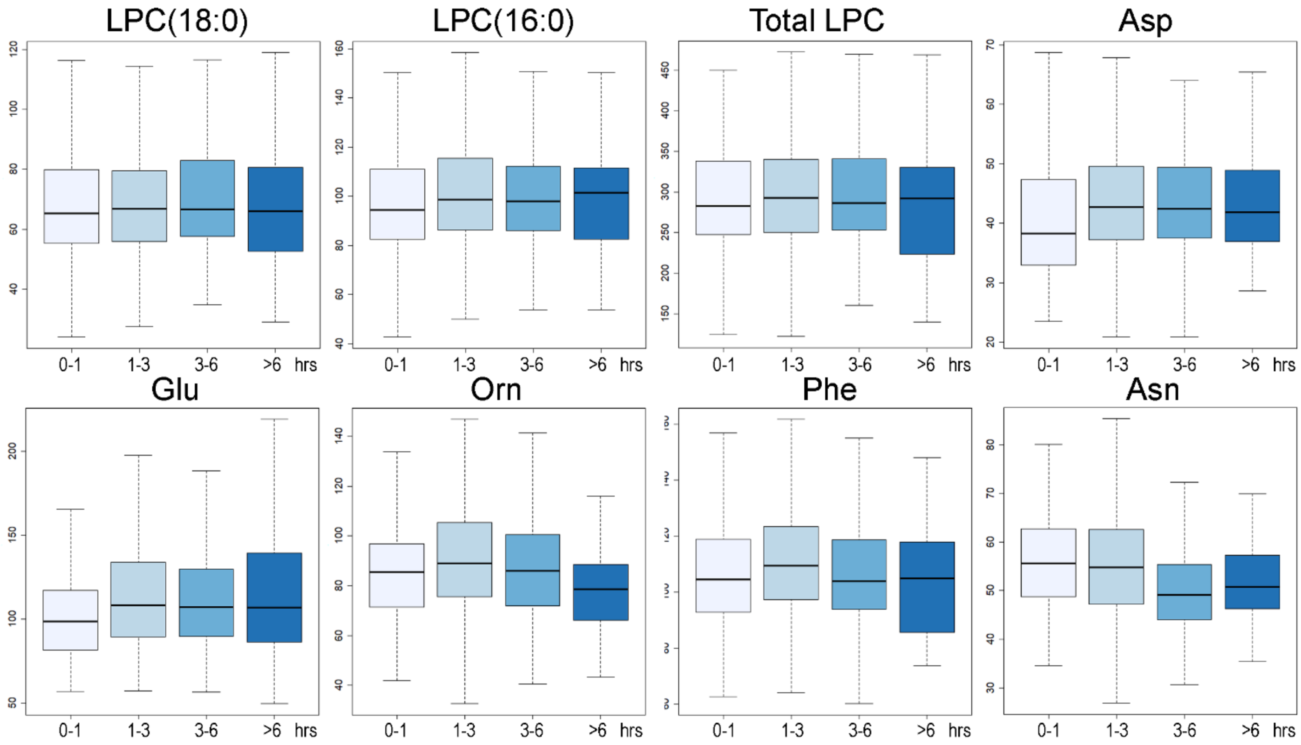

Supplementary Figure S6. The possible effect of confounding factors on serum levels of metabolites. Concentrations of selected metabolites in the sera of healthy women (participants of the HUNT2 study) donating blood after various periods of fasting (analysis based on the dataset from the report of Mrowiec et al. 2023).
